# The Roles of Adipose Tissue Macrophages in Human Disease

**DOI:** 10.3389/fimmu.2022.908749

**Published:** 2022-06-09

**Authors:** Weizheng Liang, Yanxu Qi, Hongyang Yi, Chenyu Mao, Qingxue Meng, Hao Wang, Chunfu Zheng

**Affiliations:** ^1^ Central Laboratory, The First Affiliated Hospital of Hebei North University, Zhangjiakou, China; ^2^ Department of Immunology, School of Basic Medical Sciences, Fujian Medical University, Fuzhou, China; ^3^ Department of Oral and Maxillofacial Surgery, School and Hospital of Stomatology, Cheeloo College of Medicine, Shandong University & Shandong Key Laboratory of Oral Tissue Regeneration & Shandong Engineering Laboratory for Dental Materials and Oral Tissue Regeneration, Jinan, China; ^4^ National Clinical Research Centre for Infectious Diseases, The Third People's Hospital of Shenzhen and The Second Affiliated Hospital of Southern University of Science and Technology, Shenzhen, China; ^5^ School of Engineering and Applied Science, University of Pennsylvania, Philadelphia, PA, United States; ^6^ Shenzhen Key Laboratory, Shenzhen University General Hospital, Shenzhen, China; ^7^ Department of Obstetrics and Gynecology, Shenzhen University General Hospital, Shenzhen, China; ^8^ Department of Microbiology, Immunology and Infectious Diseases, University of Calgary, Calgary, AB, Canada

**Keywords:** Adipose tissue macrophages, inflammation, obesity, diabetes, insulin resistance (IR), insulin sensitivity (IS)

## Abstract

Macrophages are a population of immune cells functioning in antigen presentation and inflammatory response. Research has demonstrated that macrophages belong to a cell lineage with strong plasticity and heterogeneity and can be polarized into different phenotypes under different microenvironments or stimuli. Many macrophages can be recruited by various cytokines secreted by adipose tissue. The recruited macrophages further secrete various inflammatory factors to act on adipocytes, and the interaction between the two leads to chronic inflammation. Previous studies have indicated that adipose tissue macrophages (ATMs) are closely related to metabolic diseases like obesity and diabetes. Here, we will not only conclude the current progress of factors affecting the polarization of adipose tissue macrophages but also elucidate the relationship between ATMs and human diseases. Furthermore, we will highlight its potential in preventing and treating metabolic diseases as immunotherapy targets.

## Introduction

Obesity is caused by the excessive accumulation of lipids in adipose tissues. In recent years, obesity has become the causing factor of many chronic diseases, including type 2 diabetes mellitus (T2DM), hypertension, cardiovascular and cerebrovascular diseases, and breast cancer, thus posing a burden on not only patients’ health and finance but also social, medical system (1–4). Apart from storing nutrients, adipose tissue is also an important immune organ containing many immune cells, among which macrophages function in maintaining immune levels. “Obesity is metabolic inflammation” was first proposed by Spiegelman in 1993 (5). It was not until 2003 that researchers discovered macrophage markers in the adipose tissue of obese animals, finding that the higher the macrophage content, the higher the obesity level of the animal (6). The traditional theory holds that macrophages in peripheral tissues are derived from monocytes in the blood (7). Visceral adipose tissue (VAT), a type of white adipose tissue (WAT), is the primary location of inflammatory response in obesity. Although many immune cells participate in the inflammatory response, adipose tissue macrophages (ATM) are considered the most important and characteristic immune cells (8). The proportion of macrophages in total cells in normal adipose tissue is only 10%, but it can reach 50% in obese people (6). Based on the difference in function and activation markers, macrophages are divided into pro-inflammatory M1 and anti-inflammatory M2, with M1 macrophages contributing mostly to the increase in obesity (8–10).

**Figure 1:**
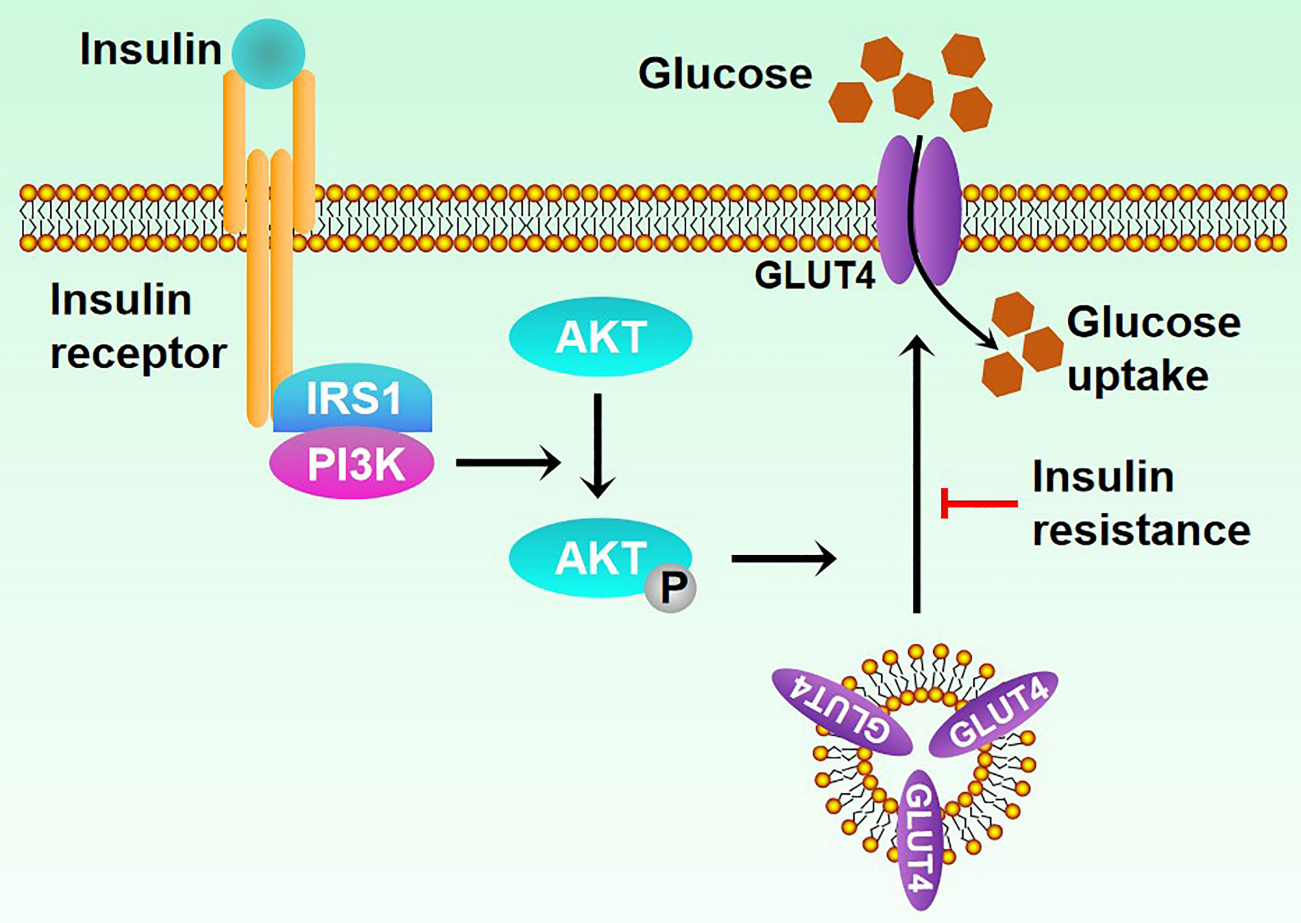
Mechanisms of insulin resistance.

T2DM poses a serious threat to human health, with 80% of its patients caused by overweight or obese. Insulin resistance (**Figure 1**), a common pathological feature of obesity, occurs when organs are insensitive to insulin stimulation, leading to high blood sugar levels, thus causing diabetes (11–13). Obesity and age-related factors are major risk factors for insulin resistance (14). Obesity stimulates NF-κB, JNK, and other signaling pathways to promote the expression of inflammatory factors, thus influencing the insulin signaling pathway and causing insulin resistance (15). Here, we will summarize the role of ATMs in human diseases and mainly focus on obesity and T2DM, thus providing new insight into the treatment of these diseases as therapeutic targets.

## Overview of ATMs

Macrophages are pivotal in the body’s immune system, and they are distributed in various tissues and organs throughout the body, including adipose tissue. Hematopoietic progenitor cells (HPCs) in the bone marrow can differentiate into monocytes upon being stimulated by various cytokines, which will transfer to VAT through the bloodstream to form ATM, thus producing corresponding inflammatory mediators and promoting HPC differentiation (16). Previous studies have shown that ATM mostly appears during embryonic development and will polarize into different phenotypes based on environment, like body weight (17, 18). When an individual is obese, macrophages are often polarized to a pro-inflammatory type, the M1 type (19, 20). With the induction of lipopolysaccharide and saturated fatty acid, M1 macrophages can activate and secrete tumor necrosis factor α (TNF-α), interleukin-6 (IL-6), interleukin-12 (IL-12), interleukin-1β (IL-1β) and other pro-inflammatory factors, leading to inflammation and insulin resistance (**Table 1**) (21). ATM manifests as an anti-inflammatory type when the individual is thin, namely the M2 type. Both M1 and M2 types have CD11b molecules on the surface. In addition, the M1 type expresses CD11c molecules, and the M2 type expresses CD206, CD301, and macrophage galactose type C-type lectin 1 specifically (29). Different from the M1 type, ATM undergoes the M2 type polarization with the induction of IL-4 and IL-13 and secretes anti-inflammatory mediators such as IL-10 and IL-1 receptor antagonists to play an anti-inflammatory role and maintain insulin sensitivity (21, 22).

**Table 1:** Properties of adipose tissue macrophages.

Properties	M1 macrophages	M2 macrophages	Refs
Inducer	TNF, LPS, Interferon	IL-4, IL-10, IL-13, IL-1β, TGFβ, LPS, Glucocorticoids	(21–28)
Secreted factors	TNF-α, IL-6, IL-12, IL-1β	Antagonists of IL-1 and IL-10 receptor	(21, 22)
Cell surface factor	CD11c	CD206, CD301	(29)

## Mechanisms of ATMs Polarization and Recruitment

### M1 Recruitment and Polarization

M1 macrophages are activated by helper T lymphocyte Th1 cytokines such as interferon, TNF, and LPS (lipopolysaccharide). The pathogenesis of obesity is closely related to the recruitment of ATMs polarized to the pro-inflammatory M1 phenotype (23). The proportion of CD11c-positive monocytes in obese patients was higher than that of normal people, which would decrease after a low-fat diet (30). Therefore, identifying the factors that can polarize ATMs to M1 and recruit macrophages to peripheral tissues in the process of obesity is of great significance for the prevention and treatment of obesity. Accumulating studies have indicated that various signaling pathways contribute to the recruitment and polarization of M1 ATMs during the progression of obesity.

MAPK (Mitogen-activated protein kinase) is a family of serine-threonine protein kinases that can be activated by different extracellular stimuli and cell adhesion, including four subfamilies: ERK, P38, JNK, and ERK5, and it is significant in the pathophysiological process of obesity (31). An early study observed the overexpression of the genes involved in p38 and JNK signaling pathways in adipose tissue of obese people (32). An animal study in mice showed that the increase of M1 ATMs proportion is achieved by increasing mRNA transcription and protein expression levels of JNK (33). In a classical study, the researchers constructed a JNK KO mouse, then fed JNK knockout mice and WT mice with a normal diet and a high-fat diet, and found that high-fat feeding increased ATMs in WT mice had few effects on KO mice (34). Moreover, the increase of macrophages in WT mice was attributed to a significant increase of M1 macrophages, while the numbers of M1 and M2 macrophages in KO mice did not show significant changes. Furthermore, the expression of M1-related genes was down-regulated, and the expression of M2-related genes was up-regulated in KO mice. These data together suggest that the activation of the MAPK signaling pathway may be related to the polarization of ATMs towards the M1 type.

Toll-like receptors are a class of innate immune receptors that are widely expressed on the surface of monocytes, macrophages, and lymphocytes, among which TLR-4 contributes to the LPS response (35). A previous study found that the transcription level of TLR4 mRNA in obese patients was remarkably higher than that in normal people, suggesting that the activation of the TLR-4 receptor may be related to the infiltration of ATMs in the process of obesity (36). Results from different labs confirmed that TLR-4 receptor deficiency reduces inflammation in adipose tissue, and TLR-4 has a positive role in the polarization of ATMs towards M1 (37, 38).

The transcription factor NF-κB is the main regulator of immune homeostasis and inflammation, discovered 30 years ago (39). Studies have demonstrated that activation of NF-κB signaling could facilitate the M1 polarization of macrophages in 3T3-L1 cell lines (40, 41). Other studies have shown that inhibiting NF-κB signaling can promote the release of IL-10 and other anti-inflammatory factors from ATMs (42). In addition, Cao et al. also observed this phenomenon in the mouse model (43). These studies strongly demonstrate that NF-κB can mediate the polarization of ATMs towards M1.

In addition to the above signaling pathways, other factors can also lead to the polarization of ATMs towards M1, including lysosomes and the AMPK signaling pathway (44–47), indicating that the polarization of ATMs towards M1 is a complex process with the coordination of multiple pathways, which needs further investigation.

### M2 Recruitment and Polarization

Th2 cytokines can activate anti-inflammatory M2 macrophage in three ways: M2a subtypes activated by IL-4 and IL-13; M2b subtype activated by immune complexes combined with IL-1β or bacterial lipopolysaccharide; M2c subtype induced by IL-10, TGFβ or glucocorticoids. During the process of inflammation resolution, M1 phenotype macrophages are polarized towards the M2 phenotype and are accompanied by the recruitment of M2 phenotype macrophages. Nuclear receptor transcription factors are significant in macrophage polarization, such as PPAR family members.

PPARγ is highly expressed in anti-inflammation macrophages and is important (48, 49). Previous research has found that activation of PPARγ can promote the conversion of M1 type macrophages to M2 type macrophages, improve insulin resistance caused by obesity, and reduce the expression of inflammatory factors (50, 51). After specific activation of PPARγ signaling in mice, it was found that the number of M1 macrophages in ATMs decreased along with the expression of M1-related genes, and the number of M2 macrophages increased, along with the expression of M2-related genes (52, 53). Furthermore, the ex vivo therapy model also demonstrated that activation of PPARγ signaling could induce the polarization of macrophages toward M2 macrophages and induce the recruitment of M2 macrophages (54). The above studies demonstrate that PPARγ is involved in ATM polarization towards M2 and M2 macrophage recruitment.

Previous studies also prove that adiponectin can promote the M2 polarization of macrophages (55). After adiponectin knockout in mice, the expression of M1-related genes was up-regulated, and the expression of M2-related genes was down-regulated. In addition, recombinant adiponectin can up-regulate the expression of M2-related genes as well (56). These results suggest that adiponectin can facilitate the polarization of adipose tissue macrophage towards M2.

IL-4 secreted by immune cells in adipose tissue can also mediate M2 polarization (24). Overexpression or knockout of IL-4 was shown to up-regulate or down-regulate the expression of M2-related genes, respectively (25). These studies demonstrate that IL-4 can mediate adipose tissue macrophage polarization toward M2. Some subsequent studies also found that cytokines such as IL-10, IL-13, and IL-33 can also mediate the polarization of macrophages towards the M2 phenotype (26–28).

## ATMs and Obesity

In the obese state, the adipose tissue is under low-intensity inflammation, and the infiltration of ATMs in it is significantly increased to a percentage of 41% compared to a normal state, accompanied by M1 polarization (6). The histological method shows that many M1 type ATMs gather around the dying adipocytes, and crown-like structures (CLSs) appear, associated with obesity-related insulin resistance (57). Further studies have shown that Mincle (macrophage-inducible C-type lectin) in ATMs is involved in the formation of CLSs, and its expression level is positively related to adipose tissue interstitial fibrosis, thus promoting liver fibrosis, progression of hepatic steatosis, and insulin resistance (58–60).

**Figure 2:**
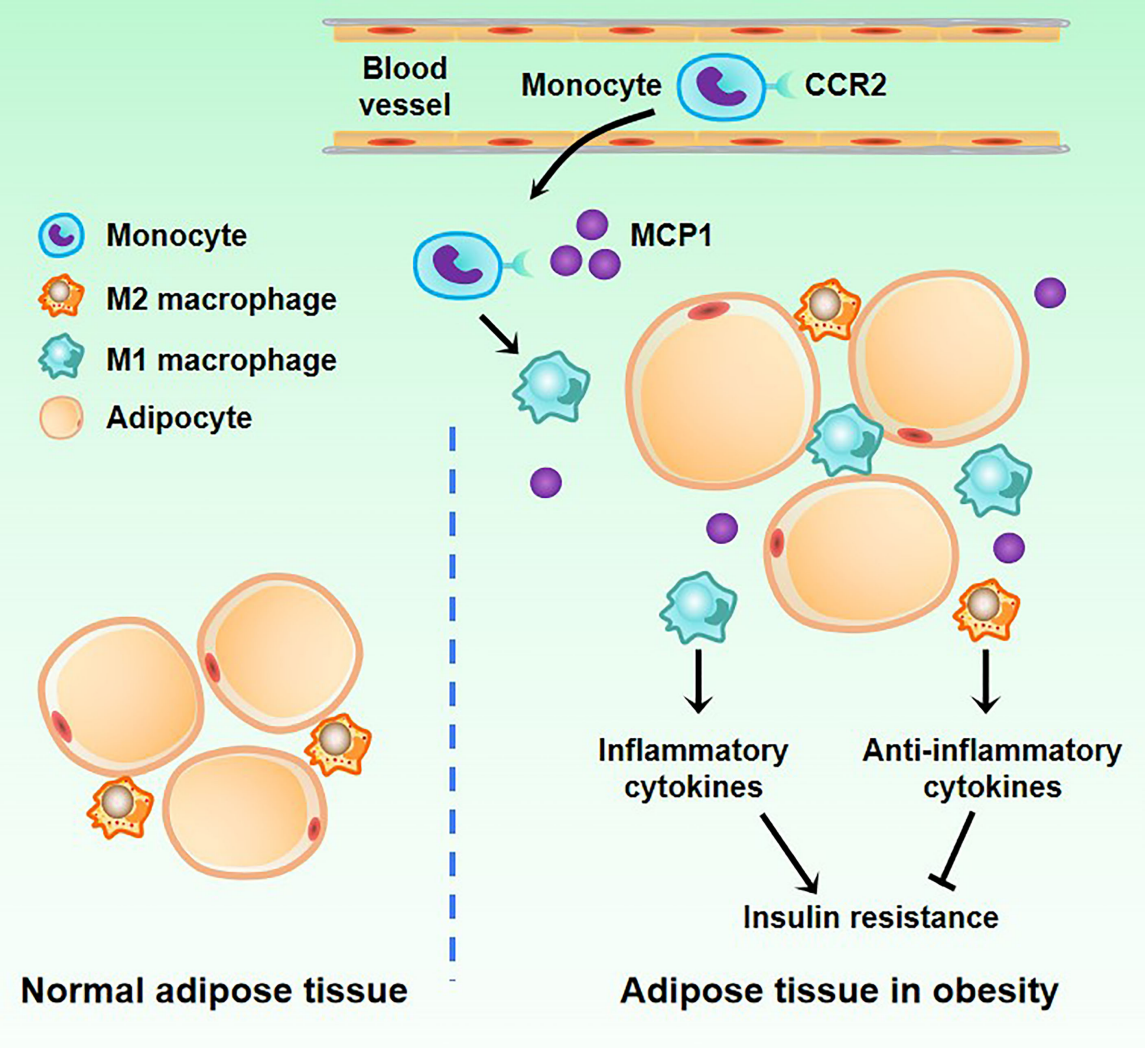
Changes of macrophages in adipose tissue in obesity. There are a small number of M2 macrophages in normal adipose tissue. When obesity occurs, blood monocytes accumulate in adipose tissue. Under the induction of the MCP1 factor secreted by adipose tissue, monocytes differentiate into M1 macrophages.

During obesity, ATM is stimulated by inflammatory factors such as IFN-γ, leukotriene B4 (LTB4), and monocyte chemoattractant protein-1 (MCP-1) released by fatty tissue, followed by M1 polarization (8, 61). Previous studies also found that the expression of IL-6, monocyte MCP-1, resistin, lipase (Adip-sin), leptin, and other factors in obese adipose tissue is up-regulated, which increases the expression of vascular endothelial cell adhesion molecules, thus recruiting monocytes in the blood, and promoting the infiltration of ATMs (**Figure 2**). Further studies confirmed that MCP-1 recruits ATMs through CCR2, while LTB4 recruits ATMs through its receptor BLT1 (62–64). The M1 type ATM secretes inflammatory factors such as IL-6, TNF-α, IL-1β, MCP-1, and PAI-1 (plasminogen activator inhibitor-1), which further increase ATM levels and maintain the M1 phenotype, thus forming a vicious circle. Studies have shown that the occurrence of various obesity-related chronic diseases, such as type 2 diabetes and atherosclerosis, are inseparable from inflammatory factors such as IL-6 and TNF-α (65, 66). In addition, in a previous study, Shimizu et al. verified that neuronal guidance molecules are also involved in the recruitment of ATMs, such as Sema3E, which can promote adipose tissue inflammation through its receptor PlexinD1 (67). Other molecules such as osteocalcin are also involved in the recruitment of ATMs and the progression of adipose tissue inflammation and may be targeted for intervention in metabolic-related diseases such as obesity (68). There is also a positive feedback loop between ATMs derived from blood monocytes and myeloid progenitors in bone marrow tissue. The NLRP3 inflammasome of ATMs is activated to stimulate myeloid progenitor cells to differentiate into monocytes and neutrophils by secreting IL-1β, and intervening in this circuit can reduce adipose tissue inflammation (16). Besides, Zhuang et al. found that miR-223 can inhibit the polarization of ATM to M1 type and ultimately inhibit the inflammatory response of adipose tissue while knocking out the miR-223 gene can aggravate the inflammatory response and increase the proportion of M1 type in ATM (69). Other studies have shown that adipose tissue inflammatory response is closely related to the β1 subunit of AMPK, and the results suggest that these molecules and enzymes may provide new entry points for future obesity treatment (70). Another study showed that IL-6 could induce the IL-4 receptor expression of ATM. ATM was significantly polarized towards the M1 type in mice that did not express the IL-6R α chain, suggesting that IL-6 may affect ATM polarization, reducing inflammation in adipose tissue (71).

Growing evidence has indicated that macrophages have a greater impact on the remodeling process of adipose tissue. First, the adipose tissue of obese animals has a higher number of macrophages, which are an important component of adipose tissue. In addition, M1 macrophages can produce some inflammatory mediators and reactive oxygen that have a certain impact on the structure and function of adipocytes. These substances will affect the normal metabolism of adipocytes and increase the release of free fatty acids (FFA), leading to increased lipotoxicity and reduction in the synthesis and secretion of adiponectin (72). Compared with normal mice, adipocyte death was significantly increased in mice with higher fat content, and a similar situation occurred in obese people, indicating that an important pathological manifestation of obesity is adipocyte death (73). A study of adipose tissue of obese patients showed that after adipocyte apoptosis, ATM surrounded it with a coronal structure, forming huge multinucleated cells, but this phenomenon was not observed in the adipose tissue of non-obese people (20). Therefore, the infiltration and activation of ATM during obesity is a powerful mechanism of adipose tissue remodeling.

In addition to the abnormal recruitment and polarization of ATMs in the adipose tissue of obese animals, their emigration is also abnormal, which is mediated by signaling molecules such as chemokines and neural guidance molecules. One previous study reported that Netrin-1 was up-regulated in ATMs of obese patients and mouse models, thus inhibiting the migration of ATMs through its receptor Unc5b (74).

M1 type ATMs are considered pro-inflammatory phenotypes in adipose tissue, and M2 type ATMs are considered anti-inflammatory phenotypes, but ATMs cannot be mechanically recognized in practice. A growing number of studies have shown that ATMs have multiple origins, with their functions spanning pure pro- or anti-inflammatory effects, and they are highly plastic and can achieve phenotypic transformation under specific circumstances, which can be therapeutic targets in the future (75, 76).

## ATMs and IR, T2MD

**Figure 3:**
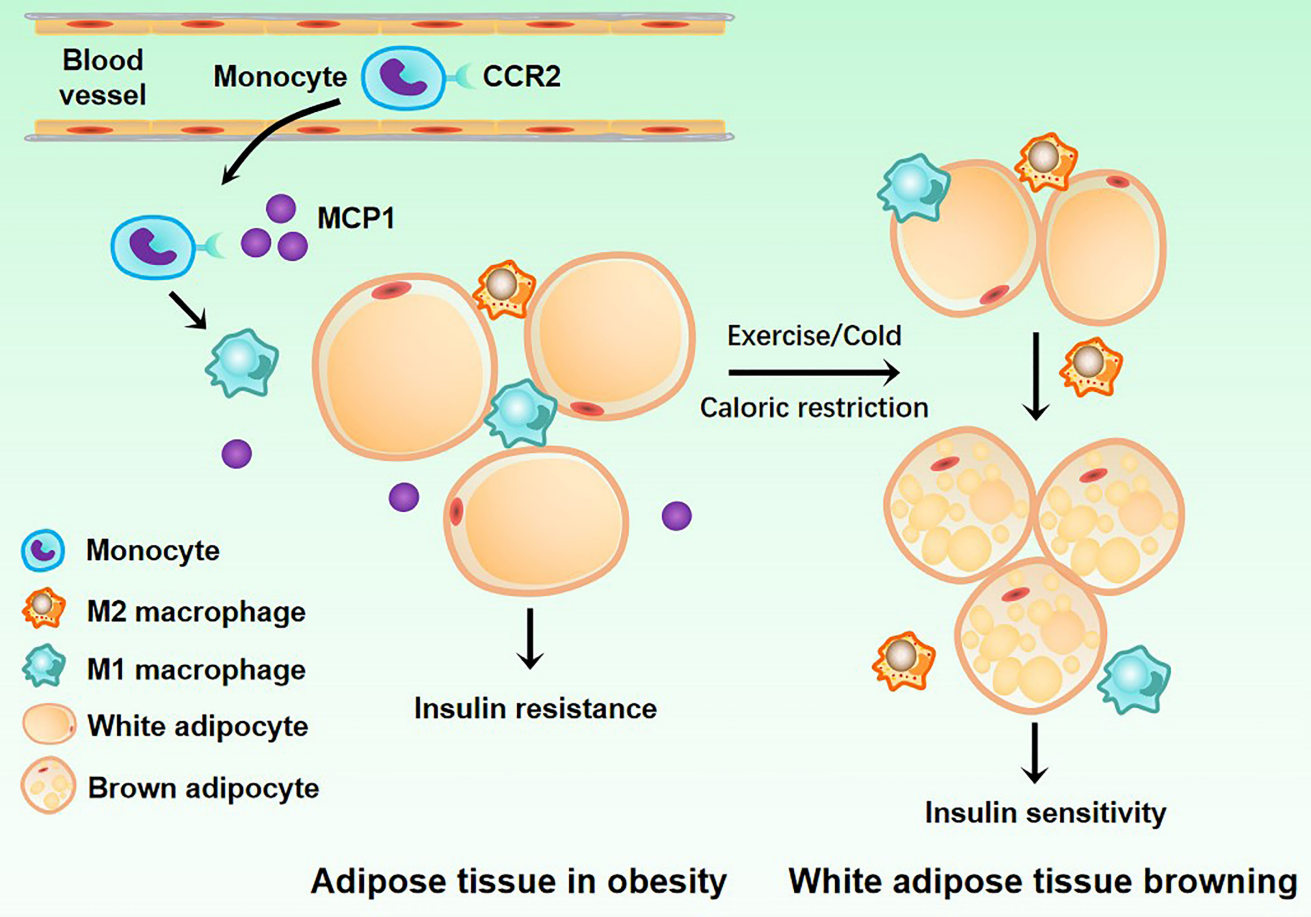
Related mechanisms of adipose tissue macrophages and type2 diabetes mellitus. White adipose tissue acquires insulin resistance under the action of M1 macrophages. When the body is exercising or dieting, M2 macrophages can induce the transformation of white adipose tissue into brown adipose tissue, allowing it to regain insulin sensitivity.

More and more studies have demonstrated that ATMs are important in IR and T2MD. Next, we will clarify the relationship between ATMs and IR and T2MD (**Figure 3**).

In a previous study, scientists demonstrated that M1 macrophages could aggravate insulin resistance, and CD11c+ cell depletion led to decreased adipose tissue inflammation and rapid normalization of insulin sensitivity (77). In another study, scientists observed that CD11c^+^ ATM ablation could reduce adipose tissue inflammatory gene expression and improve insulin resistance in the Ccr2 KO mice model (78). It can be seen that CD11c^+^ ATM infiltration of adipose tissue is one of the reasons for insulin resistance, where increasing FFAs may aggravate insulin resistance. Fetuin-A is a glycoprotein secreted by the liver, and its plasma concentration increases in obesity (79). With the mediation of Fetuin-A, FFAs can indirectly activate TLR4 of CD11c+ ATM so that nuclear factor downstream of TLR4 inhibits the phosphorylation of protein kinase β/nuclear factor-B (IKKβ/NF-κB) and c-Jun N-terminal kinase-activator protein 1 (JNK/AP-1) inflammatory signaling pathway, enhancing inflammatory gene expression and secreting more inflammatory factors like TNF-α, IL-6, and MCP-1. Some studies have found that FFAs also activate TLR2 of ATM to participate in insulin resistance (80). Physiologically, insulin mediates the tyrosine phosphorylation of the insulin receptor substrate (IRS) through the insulin receptor, thus enhancing the downstream PI3K/Akt signaling pathway, promoting glucose uptake, and exerting the hypoglycemic effect. However, activated IKKβ and JNK can cause insulin resistance through IRS serine phosphorylation and blockage of IRS tyrosine phosphorylation and the downstream PI3K/Akt pathway (81). In addition, inflammatory factors secreted by macrophages, such as TNF-α, can further activate inflammatory pathways such as IKKβ/NF-kB, JNK/AP-1, and mTOR signaling pathway, forming a vicious circle (82).

Saturated fatty acids are also involved in insulin resistance. The researchers found that knockout of the CGI-58 (comparative gene identification-58) gene in obese mouse macrophages resulted in mitochondrial dysfunction and reactive oxygen species-mediated oxidative stress in ATM, resulting in the activation of NLRP3 inflammasome and downstream caspase-1, leading to the exacerbation of insulin resistance and hyperglycemia (83). NLRP3 inflammasome is a protein complex in the cell cytoplasm, a member of the NLRs family, and its expression is increased in the adipose tissue of obese diabetic patients (84). Activated NLRP3 inflammasome and downstream caspase-1 do not affect the ratio of M1/M2 in adipose tissue but promote the secretion of IL-1β and IL-18, leading to insulin resistance (84).

Unlike the M1 type, M2 type ATMs secrete the anti-inflammatory factor IL-10, thus inhibiting inflammation and enhancing insulin resistance (85). Therefore, activating factors of M2 macrophages also indirectly affect insulin sensitivity. PPARγ is a fatty acid sensor widely expressed in M2 ATMs, which mediates the activation of monocytes’ polarization towards M2 macrophages (86, 87). With the PPARγ gene knocked out in obese mice, the expression levels of related genes in M2 type macrophages in adipose tissue decreased by 70% to 80%, while the expression levels of inflammatory genes in M1 type macrophages increased, accompanied by insulin resistance and exacerbated hyperglycemia, suggesting that PPARγ is vital in maintaining M2 macrophage phenotype and recovering insulin sensitivity (86). KLF4, as another M2-related cytokine, can synergize with IL-4 to activate STAT6 and inhibit the NF-kB signaling pathway, thus activating M2 type polarization and inhibiting M1 type polarization (88). Knockout of KLF4 in macrophages of obese mice would decrease the proportion of M2 type ATMs and worsen insulin resistance and hyperglycemia (88). In addition, compared with normal people, the expression level of KLF4 in subcutaneous adipose tissue of obese patients decreased by 50%, which may be one of the reasons for the increased M1/M2 ratio in adipose tissue.

In addition, ATM can secrete an exosome (Exos) containing microRNA (miRNA). Intravenous injection of ATM-secreted Exos (ATM−Exos) from obese mice into normal mice for 2 weeks resulted in impaired glucose tolerance and IS, suggesting the occurrence of T2DM. In contrast, when ATM-Exos from normal mice were injected into obese mice, their glucose tolerance and IS were significantly improved, and the overexpression of miR-155 in obese mice ATM-Exos inhibited the expression of its downstream IS-promoting target gene PPARγ, thereby impairing insulin signaling, leading to IR (89). Another study found that miR-29a was overexpressed in ATM-Exos of obese mice and transferred to adipocytes, cardiomyocytes, and hepatocytes, causing IR (90). ATM−Exos can be paracrine to insulin target cells, impacting intracellular insulin and glucose homeostasis. However, there are hundreds of miRNAs in ATM−Exos, and none of them affects IS alone, which may be that multiple miRNAs work together to affect adipose tissue metabolism. The above studies have shown that ATMs secrete exosomes carrying miRNAs, which can be transported to insulin target cells through paracrine or endocrine mechanisms, significantly enhancing the action of intracellular insulin, improving insulin sensitivity and overall glucose homeostasis.

Obesity is closely related to T2MD observed in clinical practice (91). Recent studies suggest that ATMs and the inflammatory response play a bridge role in this process (63, 69). The c-Jun N-terminal kinase (JNK) signaling pathway is significant in obesity-related metabolic responses. In a high-fat diet-induced obesity mouse model, although macrophage-specific JNK knockout did not affect the bodyweight of mice, it reduced ATMs infiltration and improved insulin sensitivity, and JNK knockout could inhibit the polarization of ATMs towards M1 (34). These suggest that ATMs-related inflammatory responses, rather than obesity itself, contribute to the development of obesity-related T2MD.

Further studies have shown that ATMs are involved in obesity-related T2MD by secreting cytokines such as upd3. Studies in Drosophila have shown that depletion of macrophages or macrophage-specific knockout of upd3 can inhibit the activation of the JAK-STAT signaling pathway, thus increasing insulin sensitivity without affecting body weight (92). Mincle in ATMs plays a role in the formation of CLSs and participates in obesity-related insulin resistance (58). Furthermore, the inflammatory cytokines secreted by ATMs may be causative factors leading to insulin resistance and T2MD. In addition, PAI-1 blood levels were significantly increased in obese individuals, and further studies confirmed that it was derived from ATMs stimulated by free fatty acids (93). It is worth noting that breaking the link between ATMs and NK cells, CD8^+^T cells, and myeloid progenitor cells can inhibit ATMs-mediated inflammatory response and ultimately reduce insulin resistance, which may bring light to the treatment of T2MD (16, 94, 95).

## ATMs and Clinical Therapy

Metformin is still the first-line T2MD drug especially caused by obesity. Since macrophages are involved in insulin resistance, they are likely to be ideal targets for treating metabolic diseases. The strategy is to regulate the inflammation-related signaling pathways in macrophages, thus inhibiting their polarization toward M1 and reducing macrophages’ production of inflammatory factors. Some small interfering RNAs and small molecule drugs block the activity of M1 macrophages by inhibiting NF-κB, JNK, and other signaling pathways in macrophages, reducing their infiltration in adipose tissue, thereby improving the body’s sensitivity to insulin (96, 97). Nevertheless, the models used in most studies are mice, which did not enter clinical trials.

However, most clinical research reduces the level of inflammatory factors secreted by macrophages through inflammatory factor inhibitors to treat insulin resistance. TNF-α is the first pro-inflammatory cytokine involved in insulin resistance, but limited data can show that TNF-α is involved in glucose regulation in humans. Early research suggested that short-term administration of a single TNF-α antagonist could not modulate blood glucose homeostasis (98, 99). However, 50 patients with obesity-related metabolic diseases were treated with TNF-α inhibitor etanercept for 6 months, which could significantly improve fasting blood glucose and increase adiponectin content in blood (100). The mechanism by which TNF-α inhibitors improve blood sugar still needs further investigation.

The interaction of CCR2 with its ligand MCP-1 affects monocyte migration into tissues and regulates monocyte-to-macrophage differentiation, producing pro-inflammatory cytokines and amplifying adipose tissue inflammation (20). Accumulating studies in mice have demonstrated that CCR2 selective inhibitors or CCR2/5 inhibitors can significantly improve type 2 diabetes (101–104). Combined with CCR2 inhibitors, metformin can treat diabetes by lowering blood sugar and inhibiting inflammation. A clinical trial involving 332 diabetic nephropathy patients showed that based on standard treatment, taking the CCR2 selective inhibitor, CCX140-B, could further reduce urinary protein and protect the kidneys. Compared with the placebo group, fasting blood glucose levels were significantly lower in the inhibitor group compared to the placebo group, although there was little change in HbA1C level (102). TRIM29 inhibits the secretion of IL6 and CCL2/5 in alveolar macrophages (105). CCL2/CCR2 is not the only pathway affecting the recruitment and differentiation of macrophages. The chemokine regulatory network is very complex, with CCR1-CCL3/4/5, CX3CR1-CX3CL1, and CXCR3-CXCL10 involved in macrophage differentiation. Therefore, utilizing CCR2 inhibitors to regulate macrophages to improve insulin resistance requires the support of more clinical trial data.

## Concluding Remarks and Perspectives

**Figure 4:**
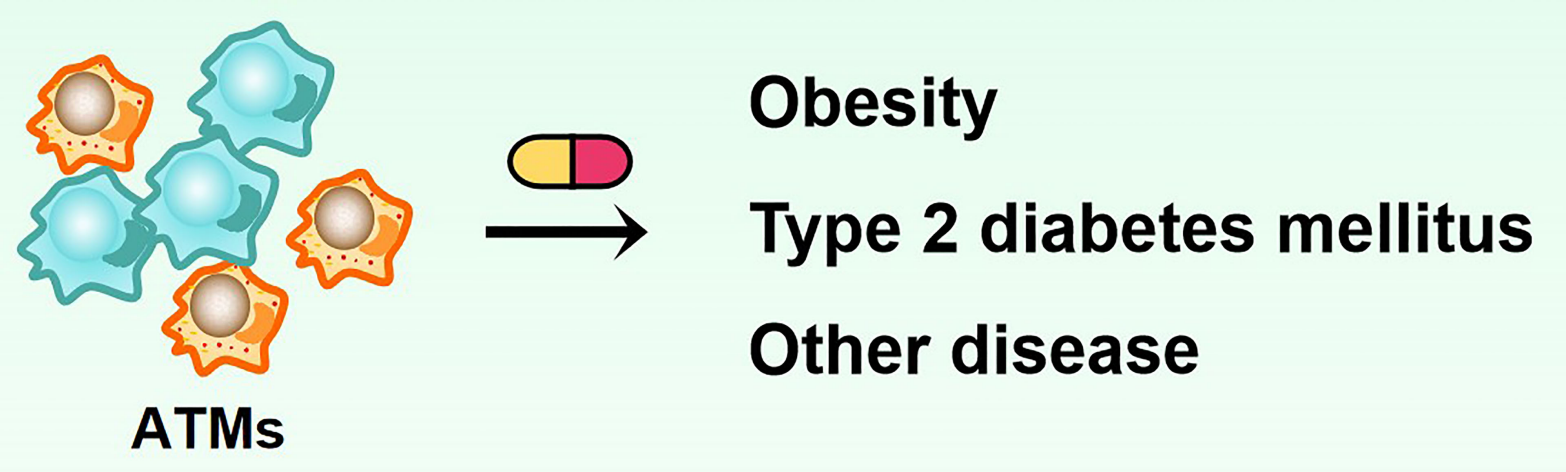
Adipose tissue macrophages can be used as a potential therapeutic target for treating obesity and diabetes.

The infiltration of pro-inflammatory macrophages in adipose tissue increases in obesity, and many inflammatory factors are secreted, resulting in adipose tissue inflammation. Inflammatory responses inhibit adipocyte insulin signaling, leading to insulin resistance. An adipose tissue macrophage is a key factor in obesity-induced insulin resistance by regulating a series of insulin-related and inflammatory factor-related signaling pathways through paracrine interactions between adipocytes and macrophages. In recent years, adipose macrophages have become a research hotspot based on their important role in insulin resistance. The in-depth study of macrophages has added new insights to the pathogenesis of metabolic diseases. In different microenvironments or under different stimuli, macrophages can show different activation modes and polarize into subtypes with different functions. Each subtype is involved in obesity, insulin resistance, T2MD, and other diseases such as atherosclerosis and severe acute pancreatitis (SAP). Therefore, the polarization direction of macrophages can be induced by regulating various factors affecting the polarization of macrophages, thereby stabilizing the balance between M1/M2 types of macrophages *in vivo*, which will make macrophages a potential new target for the treatment of metabolic diseases and bring a boon to human health (**Figure 4**).

## Author Contributions

CZ contributed directly to this review. WL wrote the preliminary version of the manuscript. YQ and WL polished the manuscript’s language and prepared figures. All authors were involved in the manuscript preparation, including figure modification, paper discussion, manuscript writing, and editing. All authors have read and approved the final manuscript.

## Funding

This work was supported by the Natural Science Foundation of Shandong Province (No. ZR2020MH190, ZR2021MH086), Medical Science and Technology Development Plans of Shandong province (No. 202008021019), and Project of Department of education of Guangdong province (No. 2021KQNCX074).

## Conflict of Interest

The authors declare that the research was conducted without any commercial or financial relationships construed as a potential conflict of interest.

## Publisher’s Note

All claims expressed in this article are solely those of the authors and do not necessarily represent those of their affiliated organizations, or those of the publisher, the editors and the reviewers. Any product that may be evaluated in this article, or claim that may be made by its manufacturer, is not guaranteed or endorsed by the publisher.
